# Coexpression network analysis of the genes regulated by two types of resistance responses to powdery mildew in wheat

**DOI:** 10.1038/srep23805

**Published:** 2016-04-01

**Authors:** Juncheng Zhang, Hongyuan Zheng, Yiwen Li, Hongjie Li, Xin Liu, Huanju Qin, Lingli Dong, Daowen Wang

**Affiliations:** 1 The State Key Laboratory of Plant Cell and chromosome Engineering, Institute of Genetics and Developmental Biology, Chinese Academy of Sciences, Beijing 100101, China; 2 The Collaborative Innovation Center for Grain Crops, Henan Agricultural University, Zhengzhou 450002, China; 3 The National Key Facility for Crop Gene Resources and Genetic Improvement, Institute of Crop Science, Chinese Academy of Agricultural Sciences, Beijing 100081, China

## Abstract

Powdery mildew disease caused by *Blumeria graminis* f. sp. *tritici* (*Bgt*) inflicts severe economic losses in wheat crops. A systematic understanding of the molecular mechanisms involved in wheat resistance to *Bgt* is essential for effectively controlling the disease. Here, using the diploid wheat *Triticum urartu* as a host, the genes regulated by immune (IM) and hypersensitive reaction (HR) resistance responses to *Bgt* were investigated through transcriptome sequencing. Four gene coexpression networks (GCNs) were developed using transcriptomic data generated for 20 *T. urartu* accessions showing IM, HR or susceptible responses. The powdery mildew resistance regulated (PMRR) genes whose expression was significantly correlated with *Bgt* resistance were identified, and they tended to be hubs and enriched in six major modules. A wide occurrence of negative regulation of PMRR genes was observed. Three new candidate immune receptor genes (*TRIUR3_13045, TRIUR3_01037* and *TRIUR3_06195*) positively associated with *Bgt* resistance were discovered. Finally, the involvement of *TRIUR3_01037* in *Bgt* resistance was tentatively verified through cosegregation analysis in a F_2_ population and functional expression assay in *Bgt* susceptible leaf cells. This research provides insights into the global network properties of PMRR genes. Potential molecular differences between IM and HR resistance responses to *Bgt* are discussed.

Powdery mildew fungi infect more than 10,000 plant species, and frequently decrease the grain yield and quality of agricultural crops^1^,^2^. Powdery mildew disease elicited by *Blumeria graminis* f. sp. *tritici* (*Bgt*) occurs worldwide in wheat crops, and can reduce grain yield by 5% to 45% depending on the severity of infestation^3^. Cultivation of resistant wheat varieties is the most economic measure for controlling the damage caused by *Bgt*^4^,^5^. In order to efficiently develop resistant varieties, it is necessary to have a detailed understanding of the genetic interactions and molecular mechanisms underlying *Bgt* resistance.

To date, more than 70 wheat genes and alleles conferring *Bgt* resistance have been identified and mapped at 49 chromosomal loci, most of which function in a race specific manner^6^. However, only four *Bgt* resistance genes (*Pm3, Pm8, Pm21* and *Pm38*) have been molecularly characterized in detail^7^,^8^,^9^,^10^, and the signaling cascades and interacting proteins required for the functions of the four genes remain largely unknown. On a genome-wide scale, the gene networks and molecular interactions involved in wheat resistance to *Bgt* are also unclear at present. The slow progress in systematically dissecting the genes and functioning molecular interactions in *Bgt* resistance may partly be caused by the high genomic complexity of the polyploid wheat species, i.e., hexaploid common wheat (*Triticum aestivum*, AABBDD, 2n = 6x = 42) and tetraploid durum wheat (*Triticum turgidum* ssp. *durum*, AABB, 2n = 4x = 28), which have often been used in past genetic and molecular studies on wheat-*Bgt* interactions. According to a chromosome-based draft sequence published recently^11^, the hexaploid genome of common wheat is approximately 17 Gb in size and contains more than 120,000 genes.

Because of its well characterized genome and the availability of abundant functional genomic resources, the model plant *Arabidopsis thaliana* has been frequently used for studying the molecular mechanisms of plant resistance to fungal pathogens including the powdery mildews *Golovinomyces cichoracearum, G. orontii* and *Erysiphe cruciferarum*^12^. Through the studies on *Arabidopsis* and similar investigations in other plant species, e.g., rice (*Oryza sativa*), tomato (*Solanum lycopersicum*) and barley (*Hordeum vulgare*), it is now clear that plants generally employ a complex, two-tiered immune system to defend against pathogen attacks, namely microbe-associated molecular pattern (MAMP)-triggered immunity (MTI) and effector-triggered immunity (ETI)^13^,^14^,^15^,^16^,^17^,^18^,^19^. The former is a basal immune response initiated after sensing MAMPs by plant cell surface located pattern-recognition receptors (PRRs)^13^,^14^,^15^,^16^. The latter is activated after recognizing pathogen encoded avirulence factors (also called effector proteins) by nucleotide-binding domain, leucine-rich repeat (NLR) proteins, which are intracellularly located plant immune receptors^17^,^18^,^19^. ETI functions by enhancing MTI, and is frequently accompanied by restricted cell death and long distance defense signaling^13^,^14^,^15^,^16^,^17^,^18^,^19^. In higher plants, typical NLR proteins have been divided into two types^17^,^18^,^19^. The first type carries a N-terminal coiled-coil (CC) domain followed by nucleotide-binding site (NBS) and leucine-rich repeat (LRR) domains (like Pm3 protein). The second type also carries NBS and LRR domains but has a TOLL/interleukin 1 receptor (TIR) domain at the N-terminus. Moreover, plant species often contain multiple genes encoding different NLR proteins^20^. For example, the model grass species rice and purple false brome grass (*Brachypodium distachyon*) carry 458 and 212 *NLR* genes, respectively^21^.

Although previous studies have suggested that the mutation of a single *NLR* gene is sufficient to turn resistance to susceptibility for a given pathogenic race^22^,^23^, an increasing number of investigations have now revealed that the coordinated function between different *NLR* genes is required for successful resistance. For example, the paired NLR decoy receptor formed by RPS4 and RRS1 proteins is needed for resistance to several bacterial and fungal pathogens of *Arabidopsis*^24^,^25^. In this receptor complex, the decoy WRKY domain of RRS1 binds the pathogen effector and initiates resistance signaling together with RPS4. Concomitant to above studies, genome-wide expression profiling and transcriptome sequencing experiments have shown that the transcript levels of multiple *NLR* genes are significantly changed after attack by a given pathogen^26^,^27^,^28^. Apart from the *NLR* genes critical for resistance signaling, many other genes are also activated in the downstream defense events. In *Arabidopsis*, it has been estimated that approximately 14% of all annotated genes may be directly related to pathogen defense^29^. In barley, a recent study using transient-induced gene silencing identified 96 genes involved in the resistance to non-adapted or adapted powdery mildew fungi^30^. Clearly, a large number of host genes take part in resistance signaling and defense processes, and they may interact in a complex manner. Because of this situation, network analysis has emerged as a valuable approach for systematically uncovering and understanding the molecular complexities of plant immunity^31^.

So far, two main types of high throughput network analysis have been applied for systematically investigating the genes involved in plant resistance responses^31^. The first one is protein-protein interactome network analysis. Two representative studies of this type concern the construction and analysis of plant-pathogen immune networks (PPIN-1 and -2) in *Arabidopsis* using yeast two-hybrid experiment^32^,^33^. The network developed is composed of four categories of proteins, i.e., pathogen effectors, effector targets in host cells, known immune proteins (including NLR, receptor like kinase, and defense proteins), and immune interactors. Through analyzing the interactome, it was suggested that many pathogen effector targets are in fact important host proteins (i.e., cellular hubs) rather than NLR proteins. This supports the guard hypothesis of plant immunity, which proposes that most NLR proteins are indirectly linked with pathogen effectors, and that the signaling function of NLRs depends on sensing the host proteins modified by pathogen effectors^32^,^33^. The immune interactors are the host genes that show strong interactions with effector targets and known immune proteins. Thus, the proteins in PPIN-1 and -2 generally represent highly connected nodes in the entire plant protein network. The functions of effector targets and immune interactors are mostly unknown at present, but Gene Ontology (GO) annotation indicates that they participate in many molecular processes, such as regulation of transcription, metabolism, nuclear targeting and phytohormone signaling, and these proteins may form a range of modules during their function in plant immune response^32^,^33^.

The second approach is based on gene coexpression network (GCN) analysis. Robust GCNs can be developed with genome scale transcriptome data, which are then used to identify the coexpressed gene sets (i.e., modules) related to a specific resistance phenotype^31^. Subsequently, cofunctional gene clusters may be defined through functional enrichment analysis of the modules with GO. A cofunctional cluster is usually composed of a hub gene and multiple neighbors, some of which may represent potentially new regulators of pathogen resistance. Using GCN analysis and combined with experimental validation, three new genes regulating the MTI response controlled by rice PRR protein XA21 have been successfully identified^34^.

In contrast to above advances, little progress has been made on understanding the genetic networks involved in pathogen resistance in non-model crop species on a genome-wide scale, especially for common wheat and its related species. Therefore, the main objective of this study was to investigate the genes and major modules involved in resistance against *Bgt* through constructing and analyzing gene coexpression networks (GCNs) using genome scale transcriptomic data. To facilitate this study, we used the diploid wheat species *Triticum urartu* (AA, 2n = 2x = 14), rather than hexaploid common wheat or tetraploid durum wheat, as the host plant. *T. urartu*, a wild grass distributed in the Fertile Crescent region, is an ancestral species of polyploid wheat, which donated the A genome to durum wheat and common wheat^35^. *T. urartu* accessions differ in their response to *Bgt* infection, and a *Bgt* resistance locus has been identified in this species^36^. In 2013, the draft genome sequence of *T. urartu* (with an estimated genome coverage of 94.33%) was reported, with 34,879 protein-coding genes annotated^37^. By analyzing the draft genome sequence, the genome size of *T. urartu* (approximately 4.94 Gb) was shown to be substantially smaller than that of common wheat (~17 Gb), but the number of *NLR* genes in *T. urartu* is considerably higher than that of rice, *B. distachyon*, maize (*Zea mays*) and sorghum (*Sorghum bicolor*)^37^. Concomitantly, a draft genome sequence was published for *Bgt*^38^. These breakthroughs provide an opportunity for the systematic study of the molecular interactions between *T. urartu* and *Bgt*. Therefore, in the present work, a set of diverse *T. urartu* accessions were screened by *Bgt* inoculation, and high-quality transcriptomic data were obtained from 20 representative lines showing immune (IM), hypersensitive reaction (HR) or susceptible responses to *Bgt* inoculation. Four GCNs were subsequently developed, whose analysis allowed identification of the genes regulated by IM or HR. The network properties of these powdery mildew resistance regulated (PMRR) genes were examined. A wide occurrence of negative gene regulation and probable involvement of three new candidate *NLR* genes in powdery mildew resistance were shown. Lastly, GO analysis was employed to infer the main biological processes enriched for the coexpressed neighbors of the three *NLR* genes in IM and HR resistance, respectively.

## Results

### Assessment of the reaction phenotypes to powdery mildew and transcriptomic data

**Figure 1:**
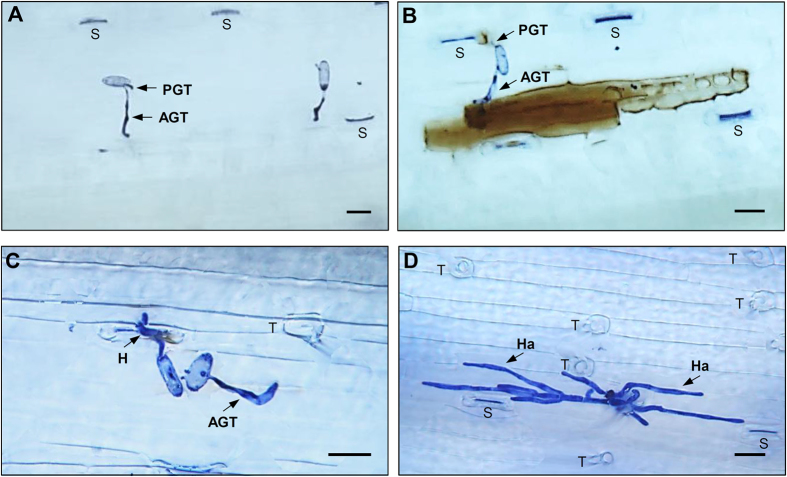
Reaction phenotypes of *T. urartu* accessions following *Bgt* inoculation. (**A**) Immune (IM) reaction to powdery mildew. *Bgt* spore germinated, but neither H_2_O_2_ accumulation nor cell death were detected in the leaf area in contact with the spore at 24 hpi. (**B**) Hypersensitive reaction (HR) to powdery mildew. *Bgt* spore germinated and elicited a strong H_2_O_2_ accumulation (brown precipitates) and cell death at 24 hpi. (**C**) Susceptible reaction to powdery mildew. *Bgt* haustorium was observed in the infected host cells at 24 hpi. (**D**) *Bgt* hyphal growth in the intercellular space of susceptible *T. urartu* accessions at 48 hpi. H_2_O_2_ accumulation and cell death were detected by staining with 3,3′-diaminobenzidine and trypan blue, respectively. AGT, appressorium germ tube; H, haustorium; Hy, hyphae; PGT, primary germ tube; S, stomata; T, trichome. Bar indicates 25 μm.

A total of 147 *T. urartu* accessions were inoculated with *Bgt*. Fourteen lines showed an immune (IM) type of resistance response. In these lines, *Bgt* spores germinated and produced primary germ tube (PGT) and an appressorium germ tube (AGT) at 4 h post inoculation (hpi), and the adjacent host cell exhibited little hydrogen peroxide (H_2_O_2_) accumulation and no cell death at 24 hpi (Fig. 1A). Fifty accessions displayed a hypersensitive reaction (HR) type of resistance response to *Bgt*. In these 50 accessions, *Bgt* spores germinated and produced PGT and AGT similar to those observed in the IM accessions at 4 hpi, but the infected cell accumulated H_2_O_2_ and underwent cell death at 24 hpi (Fig. 1B). In both IM and HR accessions, no *Bgt* hyphal growth in host intercellular space was observed at 48 hpi. In contrast, 83 *T. urartu* accessions exhibited a susceptible reaction following *Bgt* inoculation, with fungal haustoria and strong hyphal growth in the host intercellular space observed at 24 and 48 hpi, respectively (Fig. 1C,D). These observations indicated that, in *T. urartu*, the main molecular events determining IM or HR responses to *Bgt* occurred quite early, and many of them were accomplished by 24 hpi.

Subsequently, RNA-sequencing (RNA-seq) was conducted for five IM, 11 HR, and four susceptible *T. urartu* accessions using RNA samples extracted from the leaves collected at 0 (harvested prior to *Bgt* inoculation), 4 and 24 hpi, respectively. The latter two sampling time points were selected for this study based on the data presented above. Most of these accessions were from different locations of Fertile Crescent region (Table S1), thus likely represented different genotypes. As an additional control, RNA-seq was also carried out with the RNAs prepared from *Bgt* hyphae and spore materials. After adaptor sequence trimming and low-quality read filtering, high-quality reads were obtained for each *T. urartu* sample (100 bp paired-end, 61.6 million on average) (Table S1). For all 60 pair-end *T. urartu* libraries, approximately 74.1% of the high-quality reads could be aligned to *T. urartu* reference genome sequence. Those reads were estimated to cover about 45 times of *T. urartu* transcriptome assuming a transcriptome size of 0.75 Gb (15% of the 4.94 Gb *T. urartu* genome). Of the aligned reads, approximately 88.3% were mapped to unique loci, with the remaining to multiple loci (Table S1). RNA-sequencing of *Bgt* materials yielded 57,218,644 high-quality fungal reads, and 81.6% of them could be mapped to the reference genome sequence of *Bgt* (isolate 96224)^38^, with the uniquely mapped reads being 78.2% of the total. After comparing *T. urartu* reads with those of *Bgt*, the 60 *T. urartu* transcriptomic datasets were each shown to contain a low percentage (<0.2%) of fungal reads (Table S1).

### Construction of GCNs and identification of powdery mildew resistance regulated genes

After removing fungal reads, the 60 clean datasets were used to determine gene expression level by mapping to the 34,879 protein-coding genes of *T. urartu* reference genome. The fold change logarithm value for the inoculated (4 or 24 hpi)/uninoculated (0 hpi) was calculated for each expressed gene with the expression data from multiple *T. urartu* accessions, and used to construct scale-free GCNs by weighted gene correlation network analysis (WGCNA) as previously described^39^,^40^. WGCNA is a well-established method for constructing GCNs from mRNA expression data, and it considers not only the coexpression pattern between two genes, but also the overlap of neighboring genes. A weighted network retains more information and is more robust and accurate than an unweighted one in network analysis^40^,^41^. In total, four GCNs were developed, named as I4 and I24 (for IM) and H4 and H24 (for HR). The total nodes (genes) were 17,362 in I4 and H4, and 15,997 in I24 and H24, and the total edges varied substantially among the four GCNs, with the highest occurring in I4 and the lowest in H4 (Table 1). The two sets of GCNs provided the basis to investigate the genes regulating IM or HR resistance.

**Figure 2:**
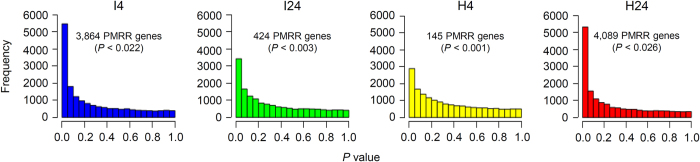
Identification of PMRR genes in the I4, I24, H4 and H24 GCNs. The *P-*value distributions of the correlations between the expression levels of *T. urartu* genes and *Bgt* resistance responses in each GCN are shown. The PMRR genes were selected using a fixed FDR level (<0.1). The number of PMRR genes thus identified and the corresponding unadjusted *P*-value are displayed for each GCN.

As a first step, the number of PMRR genes was identified in each GCN. Here PMRR genes were defined as the ones whose mRNA expression levels were significantly correlated with the resistance either positively or negatively [false discovery rate (FDR) < 0.1]. The PMRR genes thus identified for I4, I24, H4 and H24 were 3,864 (*P* < 0.022), 424 (*P* < 0.003), 145 (*P* < 0.001) and 4,089 (*P* < 0.026), respectively (Fig. 2). The number of edges for the four sets of PMRR genes differed greatly, with the highest detected for the PMRR genes in I4 and the lowest for those in H4 (Table 1). The PMRR genes shared by I4 and I24 were 314, with the total number being 3,974 (Figure S1A). For H4 and H24, the shared PMRR genes were 125, and the total number of such genes was 4,109 (Figure S1A). The PMRR genes shared between IM and HR GCNs were 2,102, and the combined PMRR genes in the four GCNs were 5,982 (Figure S1A). The PMRR genes identified above showed great robustness through the assessment of subset samples (Figure S1B).

### Identification and analysis of network hubs and hub-PMRR genes

One key property for a node in a biological network is connectivity, which reflects how frequently a node interacts with other nodes. Based on node connectivity, genes can be classified into hub (with comparatively high level of connectivity) and non-hub types. Hub genes are very important nodes, because they often encode indispensable proteins^39^,^40^,^41^. Generally, hubs represent a small proportion of the genes in a GCN, but with relatively high information exchange with other nodes.

**Figure 3:**
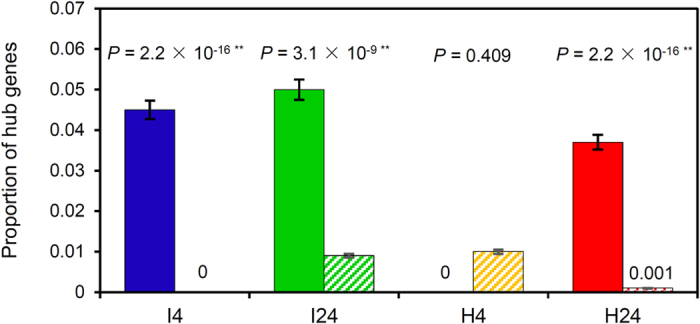
Enrichment test of PMRR genes in the hubs in I4, I24, H4 and H24 GCNs. The test was conducted using the numbers of hub genes, PMRR genes and non-PMRR genes in each GCN. Solid bars represent the proportions of hub genes among PMRR genes; striped bars represent the proportions of hub genes among non-PMRR genes. Error bars indicate ±1 s.e.m. The *P*-values shown were calculated based on Fisher’s exact test.

The connectivity distributions of all nodes were examined in each GCN (Figure S2), and 1% of nodes with the highest connectivity were defined as hub genes^42^,^43^,^44^,^45^. At this level, the numbers of hub genes in I4, I24, H4 and H24 were 174, 160, 174 and 160, respectively (Figure S2). From Fig. 3, it is clear that PMRR genes were significantly enriched in the hubs for I4 (*P* < 2.2 × 10^−16^), I24 (*P* < 3.1 × 10^−9^) and H24 (*P* < 2.2 × 10^−16^), but not H4 (*P* = 0.409). There was also significant enrichment of PMRR genes in the hub nodes for I4 (*P* < 2.2 × 10^−16^), I24 (*P* < 2.2 × 10^−16^) and H24 (*P* < 2.2 × 10^−16^) when defining the 5% of nodes with the highest connectivity as hubs (Figure S3).

The genes that were both hub and PMRR (designated hub-PMRR genes, hereafter) in I4, I24, H4 and H24 were 174, 21, 0 and 151, respectively (Figure S2, Table 1). The edges detected for the three sets of hub-PMRR genes differed substantially, with the highest number of edges occurring for the hub-PMRR genes of I4 and the lowest for the hub-PMRR genes of I24 (Table 1). I4 and I24 shared only one hub-PMRR gene, so the total number of hub-PMRR genes in the two GCNs were 194 (Figure S4). The hub-PMRR genes shared between IM (I4 and I24) and HR (H4) GCNs were 12, and the total number of hub-PMRR genes in the three GCNs were 333 (Figure S4).

The gene significance (GS) value (ranging from −1 to 1, see Methods) was investigated for each of the 333 hub-PMRR genes. Here, GS value reflected the correlation of a hub-PMRR gene expression profile with IM or HR resistance. The higher the GS value, the more significant the gene may be in *Bgt* resistance. In general, the GS values of the 333 hub-PMRR genes were higher than ±0.6 (FDR < 0.1) (Table S2). Notably, of the 333 genes, 238 (71.5%) displayed negative GS values, indicating that they were negatively correlated with *Bgt* resistance. Many of the 333 hub-PMRR genes shared identical annotations with the *Arabidopsis* genes interacting with *G. orontii* (*Gor*) effectors in PPIN-2^33^ (Table S3). For example, the genes encoding RING/U-box superfamily proteins were among both *Gor* effector-interacting *Arabidopsis* genes and our hub-PMRR genes. The hub-PMRR genes specifying RING/U-box superfamily protein generally exhibited significant GS values whether they were found in I4 or H24 GCNs (Tables S2 and S3).

### Investigation of major modules correlated with *Bgt* resistance

**Figure 4:**
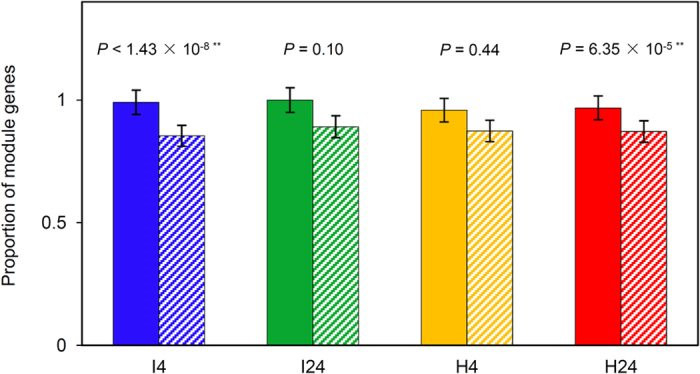
Enrichment test of PMRR genes in the modules in I4, I24, H4 and H24 GCNs. The test was executed using the numbers of module genes, PMRR genes and non-PMRR genes in each GCN. Solid bars represent the proportion of module genes among PMRR genes; striped bars represent the proportions of module genes among non-PMRR genes. Error bars indicate ± 1 s.e.m. *P*-values were calculated based on Fisher’s exact test.

Another important property of a GCN is modularity, i.e., genes that are highly interconnected within the network are usually involved in the same biological module or pathway^46^,^47^. Therefore, the number of modules was examined for each GCN constructed here, and 49, 38, 28 and 53 modules were detected in I4, I24, H4 and H24, respectively (FDR < 0.1) (Figure S5). The number of modules containing PMRR genes in the four GCNs were 50, specifically, 12 in I4, five in I24, six in H4, and 27 in H24 (Table S4). The PMRR genes in the modules of I4, I24, H4 and H24 amounted to 3,827, 424, 139 and 3,956, respectively, accounting for 99%, 100%, 96% and 97%, respectively, of the total PMRR genes in the four GCNs (Table S4). The enrichment test showed that PMRR genes tended to belong to modules, especially for I4 (*P* < 1.43 × 10^−8^) and H24 (*P* = 6.35 × 10^−5^) GCNs (Fig. 4). Simulation analysis also revealed enrichment of PMRR genes in the modules for I4 (*P* = 0) and H24 (*P* = 0) (Figure S6).

The correlations between the 50 PMRR gene-containing modules and resistance responses (IM and HR) were assessed (see Methods). Based on a combined consideration of correlation coefficient and corresponding *P-*value (<0.05), the modules significantly correlated with *Bgt* resistance were three, five, four and six for I4, I24, H4 and H24, respectively, and among the 18 significantly correlated modules, both positive and negative correlations with *Bgt* resistance were found (Table S4). To focus on the major modules, the distribution of hub-PMRR genes was investigated in the 18 modules. The hub-PMRR genes were distributed in a highly biased manner among the modules in I4, I24 and H24 GCNs, with no hub-PMRR genes observed in any of the four modules of H4 (Table S4). The first three modules (M_I4_1–M_I4_3) of I4 were considered to be the major ones because together they contained all of the 174 hub-PMRR genes identified in this GCN. Similarly, M_I24_1 (harboring 19 of the total 21 hub-PMRR genes of I24) was regarded as the major module in I24, and M_H24_1 and M_H24_2 (possessing 150 of the total 151 hub-PMRR genes of H24) as the major modules in H24 (Table S4).

### Finding of disease resistance related genes in six major modules

To gain a further understanding of the six major modules, homologs of known resistance related genes in them were investigated. In total, 139 homologs were identified, which included 31 coding for putative NLR proteins, five for pathogenesis-related (PR) proteins, 10 for mitogen-activated protein (MAP) kinases, eight for WRKY transcription factors, seven for autophagy related proteins, 50 for GTP signaling related proteins, five for glutathione S-transferases, five for pectin metabolism related proteins, 14 for peroxidases, three for chitinases, and one for isochorismate synthase (Table S5). The 139 homologs were all PMRR genes, and exhibited significant GS values. Remarkably, a large proportion of these homologs (about 75.5%) exhibited negative GS values, which was especially apparent for the genes encoding NLR, MAP kinases, WRKY transcription factor, and autophagy proteins (Table S5).

To check the reliability of the negative gene regulations noticed above, the expression profiles of seven randomly chosen homologs before and after *Bgt* inoculation of the *T. urartu* accession PI428322 (showing HR resistance to *Bgt*, Table S1) were examined by quantitative RT-PCR (qRT-PCR). The seven homologs included four coding for NLR proteins, one for a MAP kinase, one for an autophagy related protein, and one for a protein involved GTP signaling (Table S5). From Figure S7, it is apparent that the expression level of the seven homologs was all decreased at 4 and 24 h after *Bgt* inoculation. These data suggested that the negative gene regulations computed for the homologs were reliable. Lastly, for each type of homologs, there generally existed both shared and unique gene members among the major modules. For example, among the 31 *NLR* genes, ten were found in both IM and HR modules, whereas another 14 and seven were specifically present in the relevant IM or HR modules (Table S5).

### Analysis of three *NLR* genes positively correlated with *Bgt* resistance

Among the 31 *NLR* homologs, only three, *TRIUR3*_*-*_*13045, TRIUR3*_*-*_*01037* and *TRIUR3*_*-*_*06195*, exhibited positive GS values (Table S5), and were thus positively correlated with *Bgt* resistance. *TRIUR3*_*-*_*13045* was present in both the I4 module M_I4_1 and H24 module M_H24_1, whereas *TRIUR3*_*-*_*01037* and *TRIUR3*_*-*_*06195* were found in only M_H24_1 (Table S5). Considering the critical role of NLR proteins in plant disease resistance, and that only two *NLR* genes (*Pm3* and *Pm8*) controlling *Bgt* resistance have so far been molecularly characterized in wheat, the involvement of *TRIUR3*_*-*_*13045, TRIUR3*_*-*_*01037* and *TRIUR3*_*-*_*06195* in *Bgt* resistance was examined in more detail. In *T. urartu* genome assembly, the scaffolds carrying the three genes were Scaffold68689 (41.479 kb), Scaffold25403 (332.744 kb) and Scaffold75600 (115.908 kb), respectively, and the deduced products of the three genes were all CC-NB-LRR proteins (Table S6). The common wheat orthologs of the three genes were *Traes_6AS_5AEA068A7, Traes_7AL_0E46197BE* and *Traes_3AS_51B04A5B3*, respectively, and the three orthologs resided on chromosomal arms 6AS, 7AL and 3AS, respectively (Table S6). At 4 hpi, the three genes were generally and more highly expressed in the IM and HR accessions than in the susceptible ones (Figure S8). At 24 hpi, *TRIUR3*_*-*_*06195* remained more highly expressed in all resistant lines, but the expression levels of *TRIUR3*_*-*_*13045* and *TRIUR3*_*-*_*01037* decreased in some of the IM and HR accessions (Figure S8).

**Figure 5:**
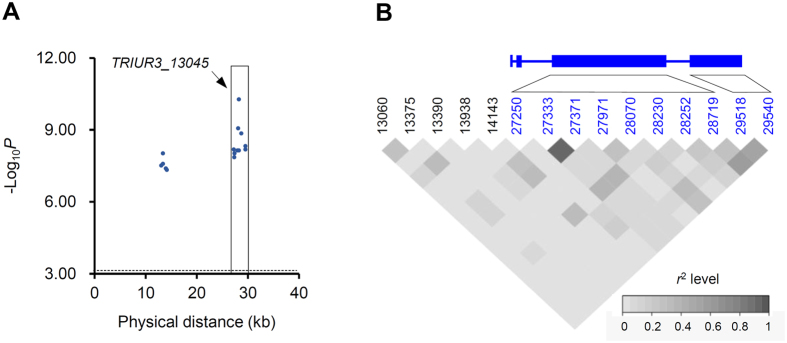
Association test of the involvement of *TRIUR3_13045* in IM resistance to *Bgt*. The test was carried out with 97 *T. urartu* accessions exhibiting IM (14) or susceptible (83) responses to *Bgt* challenge, and 15 SNPs detected in *TRIUR3_13045* genomic region. (**A**) Association plot for *TRIUR3_13045*. The 15 SNPs resided in a 40 kb genomic region. The significance threshold (*P*) was set as 0.01/total SNPs (-Log_10_*P* = 3.18, dashed line). The 10 SNPs in *TRIUR3_13045* open reading frame (ORF) (boxed) were all significantly associated with IM resistance to *Bgt*. (**B**) Linkage disequilibrium (LD) plot of the 15 SNPs used in association test. The exon (rectangle) and intron (line between rectangles) structure of *TRIUR3_13045* is shown on the top, with the 15 SNPs displayed below. The 10 SNPs, colored in blue and located in *TRIUR3_13045* ORF, were associated with IM resistance. The LD between the SNPs is measured using *r*^*2*^, which varies from 0 (white) to 1 (black).

**Figure 6:**
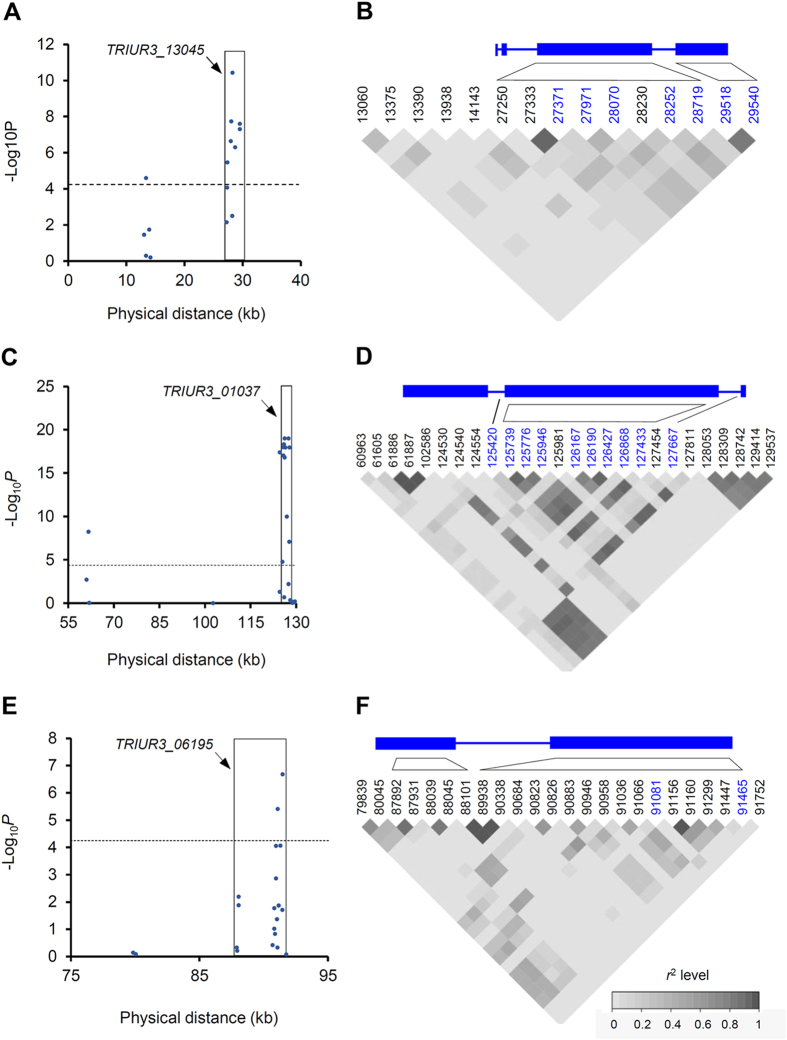
Association test of the involvement of *TRIUR3_13045, TRIUR3*_*-*_*01037* and *TRIUR3*_*-*_*06195* in HR resistance to *Bgt*. The test was carried out with 133 *T. urartu* accessions exhibiting HR or susceptible responses to *Bgt*, and various numbers of SNPs detected in *TRIUR3_13045, TRIUR3-01037* or *TRIUR3-06195* genomic regions. The significance threshold (*P*) was set as 0.01/total SNPs (−Log_10_*P* = 4.13, dashed line). To facilitate presentation, the exon (rectangle) and intron (line between rectangles) patterns of the three genes are provided. (**A**,**C**,**E**) Association plots for *TRIUR3_13045, TRIUR3-01037* and *TRIUR3-06195*, respectively. The SNPs located in the ORF of the three genes are boxed, with the ones above the threshold (dashed line) being significantly associated with HR resistance. (**B**,**D**,**F**) LD plots for the SNPs in *TRIUR3_13045, TRIUR3-01037* and *TRIUR3-06195* genomic regions, respectively. The SNPs, marked in blue and located in the respective genomic ORFs, were significantly associated with HR resistance. The LD between the SNPs is measured using *r*^*2*^ ranging from 0 to 1 (as indicated by the inset).

To investigate the participation of *TRIUR3*_*-*_*13045, TRIUR3*_*-*_*01037* and *TRIUR3*_*-*_*06195* in *Bgt* resistance, two association analysis experiments were conducted using single nucleotide polymorphisms (SNPs). The first experiment was accomplished using 97 *T. urartu* accessions showing IM (14) or susceptible (83) responses to powdery mildew for testing the involvement of *TRIUR3*_*-*_*13045* in the IM resistance. A total of 15 SNPs were observed in the Scaffold68689 carrying *TRIUR3*_*-*_*13045*, of which ten of them were located in the first and second exons of *TRIUR3*_*-*_*1304* (Fig. 5). The ten genic SNPs were all significantly associated with IM resistance to *Bgt*, with SNP-28252 showing the lowest *P*-value (5.33 × 10^−11^) (Fig. 5). Moreover, this SNP site exhibited substantial linkage disequilibrium (LD) with the other associated SNPs based on pairwise *r*^2^ levels (Fig. 5). The second experiment was executed with 133 *T. urartu* accessions showing HR (50) or susceptible (83) responses to *Bgt* for testing the involvement of *TRIUR3*_*-*_*13045, TRIUR3*_*-*_*01037* and *TRIUR3*_*-*_*06195* in the HR resistance. The genic SNPs significantly associated with HR resistance were identified for all three genes, specifically, seven for *TRIUR3*_*-*_*13045*, 10 for *TRIUR3*_*-*_*01037* and two for *TRIUR3*_*-*_*06195* (Fig. 6). The significantly associated SNPs generally exhibited medium to high levels of LD (Fig. 6). Of all three *NLR* genes, *TRIUR3*_*-*_*01037* exhibited the highest association signal (Fig. 6).

**Figure 7:**
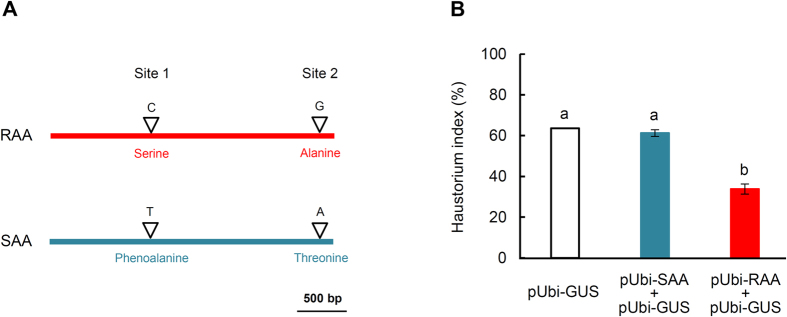
Comparative analysis of resistance associated allele (RAA) and susceptibility associated allele (SAA) of the *NLR* gene *TRIUR3_01037*. (**A**) A diagram illustrating the two SNP sites (Sites 1 and 2) in the coding region of RAA and SAA. These two SNPs were used to genotype F_2_ individuals in the cosegregation analysis. The first SNP caused a serine to phenoalanine substitution whereas the second one rendered an alanine to threonine replacement. (**B**) The effects of ectopically expressing RAA or SAA on *Bgt* haustorium index in single-cell functional expression assay. The cells were transiently transformed by pUbi-GUS alone (as control), pUbi-RAA + pUbi-GUS (for expressing RAA) or pUbi-SAA + pUbi-GUS (for expressing SAA), followed by *Bgt* inoculation. About 200 infected cells were examined for haustorium growth in each treatment. Haustorium index (mean ± SD) was calculated as the percentage of examined cells with haustorium presence. The means marked by different letters are statistically different (ANOVA, *P* < 0.05).

To further examine the involvement of *TRIUR3*_*-*_*01037* in powdery mildew resistance, a F_2_ population developed from the crossing of two *T. urartu* accessions, PI428198 and PI428322, which showed susceptibility and HR resistance to *Bgt*, respectively (Table S1), were genotyped at two selected SNP sites. These two sites differed between the resistance associated allele (RAA) and susceptibility associated allele (SAA) of *TRIUR3*_*-*_*01037*; site 1 involved a C (in RAA) to T (in SAA) change whereas site 2 was a G (in RAA) to A (in SAA) change (Fig. 7A). The SNP in site 1 caused the substitution of a serine residue (in RAA) by phenoalanine (in SAA), while that in site 2 led to the replacement of an alanine serine residue (in RAA) by threonine (in SAA) (Fig. 7A). A total of 62 F_2_ progenies were inoculated with *Bgt*, and the ratio of resistant and susceptible individuals were identified to be 47: 15 (Table S7). These data indicated that, in this F_2_ population, resistance was dominant whereas susceptibility was recessive. The genotypic data of the 62 individuals were obtained by examining SNPs at sites 1 and 2 (Fig. 7A), followed by comparison with *Bgt* reaction phenotype data. The results showed a positive match between *Bgt* reaction phenotype and *TRIUR3*_*-*_*01037* SNP genotype for the 62 individuals (Table S7). This analysis indicated that the RAA of *TRIUR3*_*-*_*01037* cosegregated with powdery mildew resistance. Subsequently, the effects of ectopic expression of RAA and SAA on *Bgt* growth in susceptible leaf cells were assessed using a transient functional expression assay (detailed in Methods). Compared with the control, expression of RAA, but not SAA, significantly decreased the growth of *Bgt* haustorium (as judged from haustorium index, Fig. 7B). The results of this functional expression assay, plus the cosegregation analysis data described above, supported a functional involvement of the RAA of *TRIUR3*_*-*_*01037* in powdery mildew resistance.

Finally, the coexpressed neighbors of *TRIUR3*_*-*_*13045, TRIUR3*_*-*_*01037* and *TRIUR3*_*-*_*06195* in the relevant modules were identified, and the main biological processes enriched by the neighbors were investigated through GO analysis (see Methods). In the IM module M_I4_1, the coexpressed partners of *TRIUR3*_*-*_*13045* were 549, 76 of which were hub-PMRR genes (Figure S9A). The biological process (BP), molecular function (MF) and cell component (CC) terms enriched by the 549 coexpressed genes were mainly related to protein translation, although the CC terms ‘plastid’ and ‘thylakoid’ were also significantly enriched (Table S8). In the HR module M_H24_1, the total number of coexpressed partners of *TRIUR3*_*-*_*13045, TRIUR3*_*-*_*01037* and *TRIUR3*_*-*_*06195* were 397, 56 of which were hub-PMRR genes (Figure S9B). The BP, MF and CC terms enriched by the 397 coexpressed genes mainly concerned photosynthesis, though the CC term ‘Golgi apparatus’ was also significantly enriched (Table S8).

## Discussion

The signaling and defense processes of plant disease resistance are highly complex and involve multiple genes. Several recent studies have elegantly demonstrated that network analysis is very effective for uncovering the genes and their interactions functioning in plant disease resistance^32^,^33^,^34^. Here GCN analysis was conducted in order to reveal the genes and major modules involved in the two types of wheat resistance responses (IM and HR) to powdery mildew. The use of transcriptomic data from multiple *T. urartu* accessions showing IM, HR or susceptible phenotypes permitted the construction of robust GCNs, thus facilitated the identification of PMRR and hub-PMRR gene sets and the major modules that were correlated with *Bgt* resistance. This provided a reliable basis for further investigations into the key mechanisms and genes that are likely important in the IM and HR responses to *Bgt*.

One of the basic questions in studying the systems biology of plant resistance to pathogen attack concerns the number of host genes regulated by disease resistance. In *Arabidopsis*, an earlier estimation suggests that about 3,000 genes (approximately 14% of all annotated *Arabidopsis* genes) may be directly involved in pathogen defense^29^. In line with this estimation, 2,043 *Arabidopsis* proteins were predicted to function in the interaction with the bacterial pathogen *Pseudomonas syringae* using domain and interolog-based computation approaches^48^. In citrus, 3,507 genes have been suggested to act in the defense response to the bacterial pathogen *Candidatus Liberibacter asiaticus* through GCN analysis^49^. In this study, the above question was approached by estimating the number of PMRR genes in four GCNs, two (I4 and I24) for IM and two (H4 and H24) for HR. For both types of responses, the GCNs developed harbored gene expression information at two different time points after *Bgt* inoculation (4 and 24 hpi), thus facilitating a more objective assessment of the genes regulated by IM or HR. Based on the total number of PMRR genes in I4 and I24 and that in H4 and H24 (Figure S1A), we suggest that in *T. urartu* the genes regulated by IM and HR resistance to *Bgt* may exceed 3,900 and 4,100, respectively. Because the total number of protein-coding genes annotated for *T. urartu* stands at 34,879 currently, it is likely that about 11%∼12% of *T. urartu* protein-coding genes may take part in its IM or HR resistance to *Bgt*. However, these values are conservative estimates because this GCN analysis could not detect the genes that did not show significant expression changes in the samples analyzed but still function in *Bgt* resistance. In addition, miRNAs and long noncoding RNAs were not considered in this GCN analysis, both of which have been shown to modulate plant pathogen resistance^50^,^51^. Nevertheless, our estimates represent a genome-wide assessment of the number of protein-coding genes likely taking part in IM or HR responses to *Bgt* in *T. urartu*. Considering the high synteny of genome organization among polyploid wheat and its diploid ancestral species and the likely conservation of the genetic mechanism of powdery mildew resistance among these species^11^,^52^, it is possible that the number of PMRR genes may exceed 8,000 in tetraploid wheat and 12,000 in hexaploid common wheat.

Previous network studies involving *Arabidopsis* and several bacterial and fungal pathogens suggest that the host genes involved in pathogen resistance are more likely to be hubs that are enriched in modules^32^,^33^,^34^. Here, the enrichment and simulation investigations revealed that the PMRR genes in only I4 and H24, but not in I24 and H4, were significantly enriched in GCN hubs and modules. Interestingly, this finding corresponds closely to the presence of more numerous PMRR and hub-PMRR genes in I4 and H24. Together, these data suggest that the PMRR genes and their interactions in I4 and H24 are probably more fundamental to the IM and HR responses to *Bgt*, respectively, when compared to those in I24 and H4. Furthermore, the 333 hub-PMRR genes identified in I4, I24 and H24 generally exhibited high GS values (Table S2), indicating that they may be strongly involved in IM or HR resistance. Supporting this proposition, the deduced products of many hub-PMRR genes resembled the proteins that have been reported to be important in the resistance response of *Arabidopsis* to the powdery mildew pathogen *G. orontii*^33^ (Table S3). According to the findings made in previous plant-pathogen interaction network studies^32^,^33^,^34^, the hub-PMRR genes identified in this study may include key players in the immunity of *T. urartu* to *Bgt*, such as the targets of *Bgt* effectors. Therefore, these hub-PMRR genes may assist in the identification of *Bgt* effector targets guarded by NLR proteins in future research. In this context, it is interesting to note that multiple hub-PMRR genes were present among the coexpressed neighbors of the three *NLR* genes likely involved in *Bgt* resistance in this study (Figure S9, see also below).

The six major modules identified in this study may reflect the main molecular interactions and processes central to IM or HR responses to *Bgt*, because 1) they generally contained a large number of PMRR and hub-PMRR genes, and 2) they all showed high level (*R* > ±0.8) and highly significant (*P* < 0.001) module-trait correlation values (Table S4). Further support for the six major modules comes from the presence of multiple types and members of resistance related gene homologs in them (Table S5). The deduced proteins of these homologs are either known to be active in resistance signaling (e.g., NLR proteins, MAP kinases, and WRKY transcription factors) or have been shown to act in defense processes (e.g., PR proteins, glutathione S-transferases, and peroxidases). Remarkably, 50 genes encoding various components of GTP signaling were present in the six modules, and all of them showed significant GS values (Table S5). This indicates that GTP signaling may play a vital role in IM and HR resistance to *Bgt*. Previous investigations in barley have identified a small GTPase (HvRacB) and its interacting proteins (HvMAGAP1 and HvELMOD_C) as important modulators of the host response to powdery mildew^53^,^54^,^55^,^56^. Recent studies in rice have established that the OsRac1 GTPase and its signaling partners, SPIN6 (a RhoGAP protein) and OsRacGEF1 (a Rho guanine nucleotide exchange factor), positively regulate immunity to both fungal and bacterial pathogens^57^,^58^. Therefore, the role of GTP signaling in *Bgt* resistance is well worth investigating. The 50 genes encoding GTP signaling related proteins, together with the major modules to which they belong, should aid such investigations.

In this research, negative correlations with *Bgt* resistance were repeatedly observed among hub-PMRR genes, major modules and resistance related gene homologs. The majority of the hub-PMRR genes (71.5%) and resistance related gene homologs (75.5%) exhibited significant negative GS values, and four out of the six major modules were negatively correlated with *Bgt* resistance. These data indicate wide occurrence of negative regulation of a large proportion of the PMRR genes in IM and HR responses to *Bgt*. Hence, it is highly possible that negative gene regulation is a key mechanism involved in *Bgt* resistance in *T. urartu*. Previous molecular genetic and network studies have also revealed the occurrence of negative gene regulation in plant-microbe interactions^59^,^60^,^61^,^62^,^63^. It has been suggested that down-regulation of gene expression facilitates effective orchestration of the key cellular processes normally under negative control (such as autophagy and cell death), fine-control of the magnitude and specificity of defense output, and proper adjustment of host physiology during and after pathogen attack^59^,^60^,^61^,^62^,^63^. Together, these actions lead to a dynamic balance between defense and growth, with efficient resource allocation and utilization. The importance of negative gene regulation in plant resistance to powdery mildews was well demonstrated by the repressive function of WRKY transcription factors in controlling the immune response mediated by barley MLA immune receptors^59^,^60^. However, judging from the large number of PMRR genes showing negative GS values in IM and HR responses, substantial efforts will be needed to fully understand the involvement of negative gene regulation in *T. urartu* resistance to *Bgt*.

Based on the GCN modeling and association analysis data gathered in this study, we propose that *TRIUR3*_*-*_*13045, TRIUR3*_*-*_*01037* and *TRIUR3*_*-*_*06195*, all showing positive GS values (Table S5), represent newly identified candidate *NLR* genes involved in *T. urartu* resistance to *Bgt*. Specifically, *TRIUR3*_*-*_*13045* may represent a potent determinant of IM resistance, whereas the three of them may all be important for HR resistance. The involvement of all three candidate *NLR* genes in HR resistance is consistent with the emerging concept on the control of plant disease resistance through molecular interactions between different NLR proteins^24^,^25^. The GCN modeling and association analysis in this study used diverse *T. urartu* accessions showing HR response to *Bgt* (11 in GCN modeling, and 50 in association analysis). This is conducive for revealing the function of multiple *NLR* genes in resistance response when compared to a typical map-based cloning approach that normally uses a limited number of genotypes. On the other hand, only *TRIUR3*_*-*_*13045* was implicated in IM resistance to *Bgt*. This may be due to the fact that relatively fewer *T. urartu* accessions exhibiting IM response were used in the GCN modeling (5) and association analysis (14), and a possibility that the molecular and functional diversities of *NLR* genes in these IM lines may be limited.

In addition to its positive association with HR response to *Bgt* (Fig. 6), functional involvement of *TRIUR3*_*-*_*01037* in powdery mildew resistance was further supported by cosegregation analysis in a F_2_ population and the expression assay of its RAA and SAA (Table S7, Fig. 7). The finding that the RAA of *TRIUR3*_*-*_*01037*, but not its SAA, could significantly decrease *Bgt* haustorium growth in a susceptible background (Fig. 7B) provides a positive indication of the action of this gene in the initiation and execution of *Bgt* resistance. However, more experiments, such as development and examination of loss-of-function mutants and transgenic wheat plants stably expressing the different alleles of *TRIUR3*_*-*_*01037*, are needed to finally validate the function of *TRIUR3*_*-*_*01037* in powdery mildew resistance.

Apart from *TRIUR3*_*-*_*13045, TRIUR3*_*-*_*01037* and *TRIUR3*_*-*_*06195*, 28 *NLR* genes exhibited negative GS values in the major modules (Table S5). The expression of multiple *NLR* genes has frequently been observed in the plants grown under normal conditions or challenged by artificial pathogen inoculation^26^,^27^,^28^. These *NLR* genes may be involved in monitoring potential pathogenic microbes in the surrounding environments, or have alternative functions in other physiological processes^32^,^64^. When encountering a strong pathogen challenge (such as artificial inoculation of *Bgt* spores), the expression of specific *NLR* gene(s) is up-regulated, with concomitant down-regulation of other *NLR* genes. Under this scenario, down-regulation of the 28 *NLR* genes may aid in the proper function of *TRIUR3*_*-*_*13045, TRIUR3*_*-*_*01037* and *TRIUR3*_*-*_*06195* in *Bgt* resistance. Further research is required to verify this hypothesis.

Based on comparisons of the 14 accessions showing IM and 50 accessions exhibiting HR, this study indicates that IM resistance arrests *Bgt* growth in the absence of host cell death (Fig. 1). This type of resistance response has also been observed and studied in other pathosystems. For example, pathogen resistance controlled by barley Mla1 and Rdg2a proteins, both of which are NLRs, occur without HR^65^,^66^. The potato NLR protein Rx confers strong resistance to potato virus x in the absence of HR^67^. Similarly, natural alleles of two *Arabidopsis* NLRs, RPS4 and RPS6, inhibit pathogen growth without involving HR^68^. More importantly, our GCN modeling suggests that IM and HR involve many differences in the expression of PMRR genes. For IM, PMRR gene expression and their interactions may mainly occur soon after infection (i.e., before 4 hpi). This is supported by the finding of substantially more PMRR genes and hub-PMRR genes in I4 rather than I24 (Table 1). On the other hand, for HR, the expression and genetic interactions of PMRR genes may largely occur at a comparatively later stage (probably later than 4 hpi, but before 24 hpi) because considerably more PMRR genes and hub-PMRR genes were detected in H24 rather than H4 (Table 1). Lastly, as discussed above, the *NLR* genes involved in IM and HR responses may be different, with one (*TRIUR3*_*-*_*13045*) implicated in IM and three (*TRIUR3*_*-*_*13045, TRIUR3*_*-*_*01037* and *TRIUR3*_*-*_*06195*) in HR.

The differential involvement of *TRIUR3*_*-*_*13045, TRIUR3*_*-*_*01037* and *TRIUR3*_*-*_*06195* in IM and HR may provide a valuable basis for further investigation of the different molecular interactions and processes in the two types of resistance responses. It is important to point out that the network characteristics of the coexpressed neighbors of *TRIUR3*_*-*_*13045* in IM differed considerably from those of *TRIUR3*_*-*_*13045, TRIUR3*_*-*_*01037* and *TRIUR3*_*-*_*06195* in HR (Table S8, Figure S9). The main biological process enriched for the coexpressed neighbors of *TRIUR3*_*-*_*13045* in IM (protein translation) was clearly different from that for the coexpressed neighbors of *TRIUR3*_*-*_*13045, TRIUR3*_*-*_*01037* and *TRIUR3*_*-*_*06195* in HR (photosynthesis) (Table S8). According to the guard hypothesis and previous studies^32^,^33^, the coexpressed neighbors of a NLR protein may contain the pathogen effector targets and the protein(s) required for the normal function of effector targets. The NLR protein monitors the binding of pathogen effector to its target, thereby controlling resistance signaling and downstream defense events. Thus, in IM, the target of *Bgt* effector may be certain component of host translation machinery that is guarded by *TRIUR3*_*-*_*13045*. On the other hand, in HR, the target of *Bgt* effector may be certain component of host photosynthesis process guarded by one or more of the three *NLR* genes (*TRIUR3*_*-*_*13045, TRIUR3*_*-*_*01037* and *TRIUR3*_*-*_*06195*).

Previous studies have shown that protein translation elongation factors of higher plants are often targeted by viral effectors, and that recessive mutations of translation elongation factors can confer strong resistance to viral pathogens^69^. Plant photosynthesis machinery, especially its oxygen evolving complex (OEC), has been shown to be required for developing HR^70^, and a bacterial pathogen effector, HopN1, targets the PsbQ protein of tomato OEC to suppress host defense^71^. At present, little has been reported on the interaction of powdery mildew effectors with host translation or photosynthesis components. The finding that photosynthesis was enriched for the coexpressed neighbors of *TRIUR3*_*-*_*13045, TRIUR3*_*-*_*01037* and *TRIUR3*_*-*_*06195* in HR, but not those of *TRIUR3*_*-*_*13045* in IM, is consistent with the observed H_2_O_2_ accumulation and presence of cell death in HR, but not IM, response (Fig. 1). Therefore, further studies of the coexpressed neighbors of *TRIUR3*_*-*_*13045, TRIUR3*_*-*_*01037* and *TRIUR3*_*-*_*06195* may reveal the roles of translation and photosynthetic processes in wheat immunity to *Bgt*.

In summary, this study has shed new light on *T. urartu* genes regulated by two types of resistance responses to *Bgt* on a genome-wide scale. The GCNs developed and the PMRR gene sets and major modules identified are useful for finding the wide occurrence of negative gene regulation, and the involvement of three new *NLR* genes, in *Bgt* resistance. The information generated has increased the understanding on IM and HR responses to *Bgt* infection, and may continue to aid further research into the molecular differences between the two types of resistance responses. This study has benefited from the lower ploidy level of *T. urartu* and the availability of a reference genome sequence for this species. These advantages, together with the fact that more and more information is becoming available for *Bgt* effector proteins^38^, lead us to suggest that *T. urartu* represents an efficient model for further and deeper systems biology studies of wheat-*Bgt* interactions.

## Methods

### Plant materials, *Bgt* inoculation and microscopic examination

A total of 147 *T. urartu* accessions collected from Armenia, Iran, Iraq, Lebanon, Syria and Turkey were subjected to two independent *Bgt* inoculation tests with the isolate E09 as previously reported^36^. In each test and for each accession, approximately 25 seeds were planted in compost in a growth chamber set at 25 °C (with a photoperiod of 18 h light/6 h dark and a relative humidity of 80%). At the two-leaf stage, 10–15 well developed seedlings were selected in each accession and inoculated with *Bgt*. The growth temperature was adjusted to 20 °C following the inoculation. The reactions to *Bgt* inoculation were examined visually, and classified into three types, IM, HR or susceptible reaction, based on the presence (or absence) of hypersensitive necrotic spots and powdery mildew colonies on the leaf surface. Highly similar results were obtained between the two separate inoculation tests. Subsequently, *Bgt* spore germination and host cell change were examined in more detail in 20 representative accessions (Table S1) showing IM, HR or susceptible responses at 4, 24, 48 and 96 hpi. Detection of H_2_O_2_ accumulation and cell death by staining with 3,3′-diaminobenzidine or trypan blue were accomplished as detailed in previous studies^72^,^73^. The stained leaf materials were examined under a compound microscope (Leica DMRE, Wetzlar, Germany), with the images collected using a digital camera (EXi Aqua, QImaging, BC, Canada).

### RNA sequencing and preparation of high-quality transcriptomic reads

The 20 *T. urartu* accessions used in transcriptome sequencing exhibited IM (5), HR (11) or susceptible (4) reactions to *Bgt* infection (Table S1). Leaf samples were collected from the 20 accessions prior to *Bgt* inoculation and at 4 and 24 hpi of *Bgt*. For each accession and at each time point, leaf samples from five uniform seedlings were combined for total RNA extraction using Illumina TruSeq RNA Sample Prep Kit (Illumina, Inc., San Diego, USA). A total of 60 paired-end libraries were constructed with Illumina Paired-End Sample Prep Kit, followed by sequencing on Illumina HiSeq 2000 platform. Adaptor sequence trimming and removal of low-quality reads were performed with the ngsShoRT algorithms^74^. A paired-end library was also constructed using the total RNA extracted from *Bgt* hyphae and spore materials collected from a susceptible *T. urartu* accession (PI428198, Table S1) at 168 hpi. The library was sequenced and high-quality reads were obtained as described above. Genome mapping of high-quality reads was executed using the software TopHat2^75^. The references used for genome mapping were either *T. urartu* draft genome assembly (http://gigadb.org/dataset/100050, for mapping the reads obtained from 60 *T. urartu* libraries) or the whole genome assembly of *Bgt* isolate 96224 (www.ncbi.nlm.nih.gov/Traces/wgs/?val=ANze&display=contigs&search=ANze01000000, for mapping the reads from *Bgt* hyphae and spore library).

While designing the transcriptome sequencing scheme described above, it was considered that the inclusion of multiple *T. urartu* accessions showing IM, HR or susceptible phenotypes could facilitate realistic estimations of gene expression levels. Therefore, no biological replicates were collected and sequenced. Previous studies have also used a similar strategy in examining gene expression levels through transcriptome sequencing^76^,^77^,^78^.

### Calculation of gene expression level and network construction

The expression level of *T. urartu* genes, measured as fragments per kilobase of transcript per million fragments sequenced, was determined using the reads with positive genome mapping by DEGseq^79^. The reference used was the coding sequence of 34,879 *T. urartu* genes (downloaded from http://gigadb.org/dataset/100050). To construct robust GCNs in this study, mRNA expression data of the 34,879 protein-coding genes in the 60 libraries were filtered in two ways. First, the genes whose expression was not detected in one of the 60 libraries were discarded. Second, the genes with aligned reads lower than 50 were also discarded to reduce background noise. After these filtering steps, 17,362 and 15,997 expressed genes were retained for constructing the GCNs at 4 and 24 hpi, respectively. The WGCNA (weighted gene correlation network analysis) R package was then used to build GCNs, investigate main network properties (hubs and modules), calculate the significance values of genes, and examine the correlations between modules and *Bgt* resistance phenotypes (IM or HR) as described in previous publications^39^,^40^.

### Identification of powdery mildew resistance regulated (PMRR) genes

The PMRR genes in the four GCNs (I4, I24, H4 and H24) were identified following the strategy reported previously^39^,^40^. The genes, whose FDR (false discovery rate)-corrected *P*-value was smaller than 0.1, were regarded as PMRR genes. The robustness of the PMRR genes thus identified was assessed using 100 random samples (each involving 75% of the PMRR genes in the relevant GCN). The hub-PMRR genes were located in two steps. First, the hub genes in each GCN were identified based on gene connectivity (i.e., sum of the weights across all the edges of a node) and the top 1% of the genes with the highest connectivity in the network were considered as hubs^39^,^40^. Second, for each GCN, the set of hub genes was compared to that of PMRR genes, leading to the identification of hub-PMRR genes. In each GCN, the enrichment of PMRR genes in hub nodes and in modules was examined by Fisher’s exact test^45^. Simulation tests for hub and module enrichment of PMRR genes were accomplished as described previously^45^.

### Search of homologs of known resistance related genes

The PMRR genes, which were detected in the 6 major modules (Table S4), were selected out from the 34,897 protein-coding gene set of *T. urartu*^37^, and their annotations were carefully checked. They were then subjected to a text search using 11 plant disease resistance associated keywords, including “NLR protein”, “pathogenesis-related (PR) protein”, “mitogen-activated protein (MAP) kinase”, “WRKY transcription factor”, “autophagy related protein”, “GTP signaling”, “glutathione S-transferase (GST)”, “isochorismate synthase”, “chitinase”, “pectin metabolism”, and “peroxidase”. Positive hits were identified based on the presence of key word in the annotation, and a GS (gene significance) value was computed for each hit as described above.

### Investigations of TRIUR3_-_13045, TRIUR3_-_01037 and TRIUR3_-_06195

The scaffolds (i.e., Scaffold68689, Scaffold25403 and Scaffold75600) carrying the three *NLR* genes (*TRIUR3*_*-*_*13045, TRIUR3*_*-*_*01037* or *TRIUR3*_*-*_*06195*) were found in *T. urartu* draft genome assembly (http://gigadb.org/dataset/100050). Because the scaffolds in *T. urartu* draft genome sequence were not completely ordered along chromosomes, the orthologs of the three *NLR* genes were identified in the draft genome assembly of common wheat (https://urgi.versailles.inra.fr/download/iwgsc/) by a blastN search, with positive hits judged by identity (>90%) and coverage (>90%) values. Hierarchical clustering of the expression data of the three *NLR* genes at 4 and 24 hpi of *Bgt* was generated using the software Cluster 3.0^80^.

To evaluate the involvement of *TRIUR3*_*-*_*13045, TRIUR3*_*-*_*01037* and *TRIUR3*_*-*_*06195* in *Bgt* resistance, two sets of SNP marker based case/control type of association test were performed using PLINK^81^. To facilitate the finding of SNPs, RNA-seq was carried out for 127 more *T. urartu* accessions with total RNA samples extracted from *Bgt* inoculated leaf materials as described above. This led to the availability of transcriptomic data for all 147 *T. urartu* accessions that had been evaluated for *Bgt* response in this research. In the first test, the participation of *TRIUR3*_*-*_*13045* in IM response was examined. The 14 *T. urartu* accessions showing IM response to *Bgt* were cases whereas the 83 susceptible accessions were controls. In the second test, the involvement of all three *NLR* genes in HR response was examined. The 50 *T. urartu* accessions displaying HR after *Bgt* infection were cases whereas the 83 susceptible accessions were controls. SNPs for the three scaffolds carrying *TRIUR3*_*-*_*13045, TRIUR3*_*-*_*01037* or *TRIUR3*_*-*_*06195* were called by jointly using TopHat2 (for mapping transcriptomic reads) and SAMtools (for nucleotide variant detection)^82^. The SNPs found were 15 for Scaffold68689, 92 for Scaffold25403, and 28 for Scaffold75600. The significance threshold of the association tests was set as 0.01/total SNPs. The total SNPs used for calculating significance threshold in the first and second association tests were 15 and 135, respectively. The LD plots of SNPs were drawn using the LDheatmap package^83^.

### Quantitative RT-PCR assay

The coding sequence of the seven genes, which were randomly chosen to verify the negative regulations computed for resistance related gene homologs by qRT-PCR (Table S5), was downloaded from http://gigadb.org/dataset/100050. Specific nucleotide primers (Table S9) were designed for each of the seven genes using the software Primer3 (Primer3.ut.ee). Fourty seedlings of PI428322 (with HR resistance to *Bgt*, Table S1) were inoculated with *Bgt* isolate E09 as described above. Three samples were collected at 0, 4 and 24 hpi, respectively, with each sampling involving 6 different seedlings. The extraction of total RNAs, preparation of cDNAs, and execution of qRT-PCR were conducted following a previous study^84^. A common wheat *actin* gene (GenBank accession AB181991) was used as the internal reference for qRT-PCR. The assay was conducted twice using independent biological replicates.

### Cosegregation analysis of *TRIUR3_01037* in F_2_ population

The *Bgt* reaction phenotype of the 62 F_2_ individuals (Table S7) was identified by inoculation with *Bgt* isolate E09 as described above. For genotyping the 62 individuals using the SNPs at sites 1 and 2 (Fig. 7a), amplicons containing the SNP at site 1 or 2 were obtained by genomic PCR, followed by DNA sequencing. The amplicon sequences were then analyzed to determine the genotypes of different F_2_ plants. The primers used for obtaining the amplicons are listed in Table S9.

### Single-cell functional expression assay

The single-cell transient functional expression assay was conducted essentially as described^59^. The cDNA coding region of *TRIUR3_01037* was amplified from PI428198 and PI428322, respectively, with the primers listed in Table S9. The resultant cDNA sequences, designated as RAA and SAA, respectively, were confirmed to be error-free by DNA sequencing. They were then cloned into a plasmid vector driven by the maize polyubiquitin promoter, resulting in the expression constructs pUbi-RAA and pUbi-SAA, respectively. A β-glucuronidase (GUS) reporter gene construct (pUbi-GUS) was also used in this assay, whose expression facilitated the visualization of transformed cells^59^.

The RAA and SAA constructs were each mixed with pUbi-GUS at a 1:1 molar ratio for coating the DNA microcarrier. As control for the assay, only pUbi-GUS was used for coating the DNA microcarrier. The plasmid DNA coated on the microcarrier was delivered into the leaf epidermal cells of the *T. urartu* accession PI428252, which was susceptible to *Bgt* isolate E09, through particle bombardment. The leaf segments were inoculated with E09 conidial spores at 4 h post bombardment. At 48 hpi of *Bgt*, the leaf segments were stained in a β-glucuronidase staining solution for 8 h at 37 °C, followed by destaining for 72 h at 25 °C. Before mounting for microscopic examination, the leaf segments were stained with Coomassie blue to visualize *Bgt* structures. For assessing the effects of each construct (pUbi-RAA, pUbi-SAA or pUbi-GUS), approximately 200 cells (in 6 to 8 leaf segments) with GUS signals and *Bgt* mycelia were examined for haustorium growth, with haustorium index calculated as the percentage of examined cells with haustorium presence^59^.

### Gene ontology analysis of the coexpressed neighbors of *NLR* genes

The coexpressed interactors of *TRIUR3*_*-*_*13045* in IM and all three *NLR* genes in HR were extracted from relevant GCNs (I4 or H24) and visualized using Cytoscape^85^. To aid GO enrichment analysis of the extracted neighbors, GO term annotation of *T. urartu* genes was improved using Blast2GO^86^ and InterProScan^87^. This led to the assignment of GO terms to 25,437 genes, accounting for 73% of the 34,879 genes annotated by *T. urartu* draft genome sequence. The enrichment analysis was subsequently conducted using agriGO (http://bioinfo.cau.edu.cn/agriGO/)^88^. Only the terms with a significance level lower than 0.05 and a minimum of 5 annotated genes in the input list were selected for further analysis.

## Additional Information

**Accession codes:** The transcriptomic data generated for 20 *T. urartu* accessions at 0, 4 and 24 hpi of *Bgt* have been submitted to the BioProject database of National Center for Biotechnology Information (accession number PRJNA289598).

**How to cite this article**: Zhang, J. *et al*. Coexpression network analysis of the genes regulated by two types of resistance responses to powdery mildew in wheat. *Sci. Rep.* **6**, 23805; doi: 10.1038/srep23805 (2016).

## Supplementary Material

Supplementary Information

Supplementary Dataset 1

Supplementary Dataset 2

Supplementary Dataset 3

Supplementary Dataset 4

Supplementary Dataset 5

Supplementary Dataset 6

Supplementary Dataset 7

Supplementary Dataset 8

Supplementary Dataset 9

## Figures and Tables

**Table 1:** Construction of GCNs and analysis of PMRR and hub-PMRR genes.

GCNs	Total nodes	Total edges	PMRR genes		Hub-PMRR genes	
			Nodes	Edges	Nodes	Edges
I4	17,362	84,781,451	3,864	43,149,830	174	2,363,001
I24	15,997	55,685,354	424	4,347,422	21	232,928
H4	17,362	16,910,530	145	202,415	0	0
H24	15,997	49,927,774	4,089	30,469,907	151	1,668,098
