# Supplementary material for: Coexpression network analysis of the genes regulated by two types of resistance responses to powdery mildew in wheat
Source: Sci Rep. 2016 Apr 1;6:23805. doi: 10.1038/srep23805 (PMC4817125; doi:10.1038/srep23805)
Supplement: Supplementary Information [file srep23805-s1.pdf]

Coexpression network analysis of the genes regulated by two types of resistance responses to powdery mildew in wheat

Juncheng Zhang[1], Hongyuan Zheng[2], Yiwen Li[1], Hongjie Li[3], Xin Liu[1], Huanju Qin[1], Lingli Dong[1*] & Daowen Wang[1,2*]

[1]The State Key Laboratory of Plant Cell and chromosome Engineering, Institute of Genetics and Developmental Biology, Chinese Academy of Sciences, Beijing 100101, China. [2]The Collaborative Innovation Center for Grain Crops, Henan Agricultural University, Zhengzhou 450002, China. [3]The National Key Facility for Crop Gene Resources and Genetic Improvement, Institute of Crop Science, Chinese Academy of Agricultural Sciences, Beijing 100081, China.

Correspondence and requests for materials should be addressed to L.D. (email lldong@genetics.ac.cn) and D.W. (email dwwang@genetics.ac.cn)

Figure S1. Overlapping and robustness of the PMRR genes identified in I4, I24, H4 and H24 GCNs. (**A**) Venn diagram analysis of the PMRR genes identified in four GCNs. (**B**) Distributions of the proportions of PMRR genes showing significant expression-*Bgt* resistance correlation in 100 randomly sampled subsets in I4, I24, H4 or H24. The grey line marks the average proportion of PMRR genes across the 100 sample subsets in each GCN.

Figure S2. Connectivity distributions of the genes in I4, I24, H4 and H24 GCNs and definition of hub and hub-PMRR genes. In each GCN, connectivity was calculated for both PMRR and non-PMRR gene nodes, with the PMRR gene nodes shown in grey. The top 1% of nodes with the highest connectivity was defined as hubs. The hub-PMRR genes were revealed by comparing PMRR and hub gene nodes.

Figure S3. Enrichment test of PMRR genes in the hubs defined by adjacency connectivity (5% cutoff) in I4, I24, H4 and H24 GCNs. The test was accomplished using the numbers of hub genes, PMRR genes and non-PMRR genes in each GCN. Solid bars represent the proportions of hub genes among PMRR genes; striped bars represent the proportions of hub genes among non-PMRR genes. Error bars indicate ± 1 s.e.m.. The *P*-values shown were calculated based on Fisher's exact test.

Figure S4. Overlapping of the hub-PMRR genes identified in I4, I24 and H24 GCNs.

Figure S5. Modules in I4, I24, H4 and H24 GCNs. The modules were defined through weighted gene co-expression network analysis, and represented by colorful bands. The number of modules in each GCN is given in the parentheses.

Figure S6. Simulation test of the enrichment of PMRR genes in the modules in I4, I24, H4 and H24 GCNs. The test was conducted using a random set of genes selected from non-PMRR genes as pseudo-PMRR genes. The number of genes in the

randomly selected set was equal to the actual count of the PMRR genes in a given GCN. For this simulation test, the module genes were defined at two variable FDR levels: < 0.05 (top panel) and < 0.01 (bottom panel). Solid bars represent the proportions of module genes among pseudo-PMRR genes; striped bars represent the proportions of module genes among non-PMRR genes. Error bars indicate ± 1 s.e.m.. The *P*-values displayed were calculated based on Fisher's exact test.

Figure S7. Verification of the negative regulations computed for resistance related gene homologs by qRT-PCR assay. Seven genes (*TRIUR3_21497*, *TRIUR3_05259*, *TRIUR3_02996*, *TRIUR3_07996*, *TRIUR3_09881*, *TRIUR3_19436* and *TRIUR3_02468*, Table S5) were randomly chosen as representatives for the assay. Leaf samples were collected from the seedlings of PI428322 (with HR resistance to *Bgt*, Table S1) at 0, 4 and 24 hpi of *Bgt* inoculation. The expression profiles of the seven genes at the three time points were examined by qRT-PCR using gene specific primers (Table S9). A common wheat *actin* gene (GenBank accession AB181991) was used as the internal reference for the assay. To facilitate comparison, the relative expression level at 0 hpi was set as 1, and those at 4 and 24 hpi were normalized against 1. The data shown were representative of two different assays using independent biological replicates.

Figure S8. Expression changes of *TRIUR3_13045*, *TRIUR3_01037* and *TRIUR3_06195* in 20 *T. urartu* accessions at 4 and 24 hpi of *Bgt*. The 20 accessions exhibited susceptible, immune or hypersensitive reaction phenotypes to *Bgt*. The heat map was generated from hierarchical clustering of logarithmic fold change values of the expression of the three genes. Each fold change value was calculated by comparing to the expression level at 0 hpi. The up- and down-regulations of gene expression are indicated by red and green colors, respectively.

Figure S9. Analysis of the coexpressed neighbors of *TRIUR3_13045*, *TRIUR3_01037* and *TRIUR3_06195*. The coexpressed neighbors of the three genes were extracted from relevant GCNs, and displayed as blue (hub gene) and grey (non-hub gene) triangles in the graphs shown. (**A**) The coexpressed neighbors of *TRIUR3_13045* in IM resistance. These neighbors, 549 in total and including 76 hub genes, were extracted from I4 GCN. (**B**) The coexpressed neighbors of *TRIUR3_13045*, *TRIUR3_01037* and *TRIUR3_06195* in HR resistance. The 397 neighbors, including 56 hub genes, were extracted from H24 GCN.

Table S1. Summary of RNA-seq data for 20 different *T. urartu* accessions.

Table S2. Lists of hub-PMRR genes identified in I4, I24 and H24 and 333 hub-PMRR genes in the three GCNs.

Table S3. Some annotations shared by hub-PMRR genes and the genes interacting with *G. orontii* effectors in *Arabidopsis* PPIN-2.

Table S4. Analysis of the 50 modules containing PMRR genes in I4, I24, H4 and H24 GCNs.

Table S5. Homologs of plant disease resistance related genes found in the six major modules in I4, I24 and H24 GCNs.

Table S6. Some properties of the three *NLR* genes positively correlated with *Bgt* resistance.

Table S7. *Bgt* reaction phenotypes and *TRIUR3_01037* SNP genotypes in 62 F2 individuals.

Table S8. GO terms enriched for the coexpressed neighbors of the three *NLR* genes involved in *Bgt* resistance.

Table S9. Oligonucleotide primers used in this study.

Figure S1

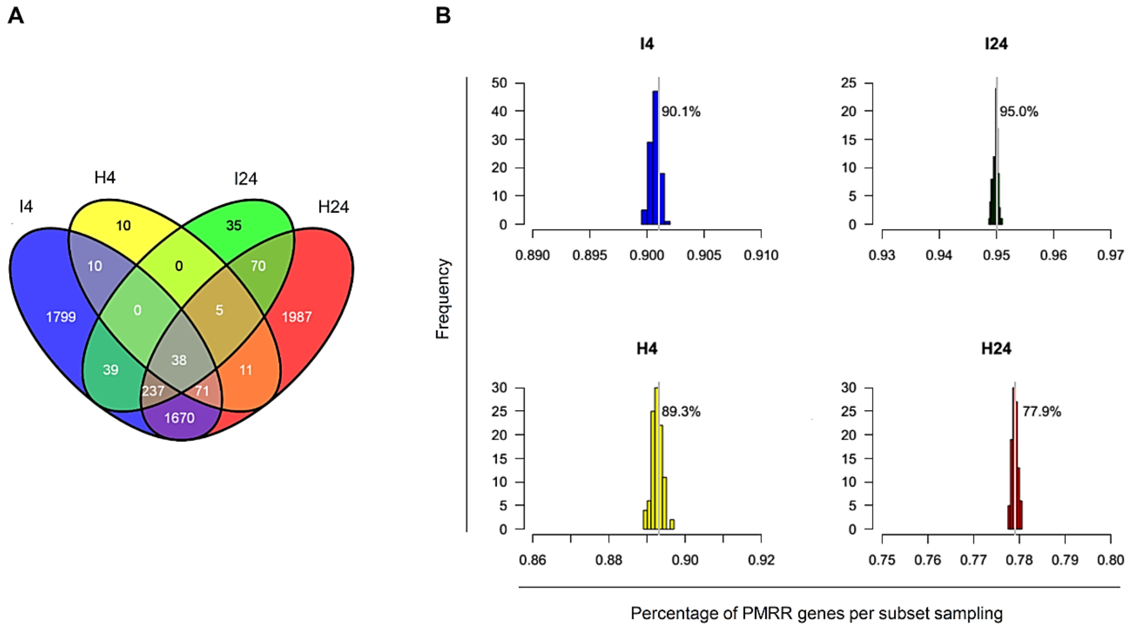
A
I4
H4
I24
H24
10
35
10
0
70
1799
0
5
1987
38
39
11
237
71
1670
B
I4
90.1%
0.890
0.895
0.900
0.905
0.910
I24
95.0%
0.93
0.94
0.95
0.96
0.97
H4
89.3%
0.86
0.88
0.90
0.92
H24
77.9%
0.75
0.76
0.77
0.78
0.79
0.80
Frequency
Percentage of PMRR genes per subset sampling

Figure S2

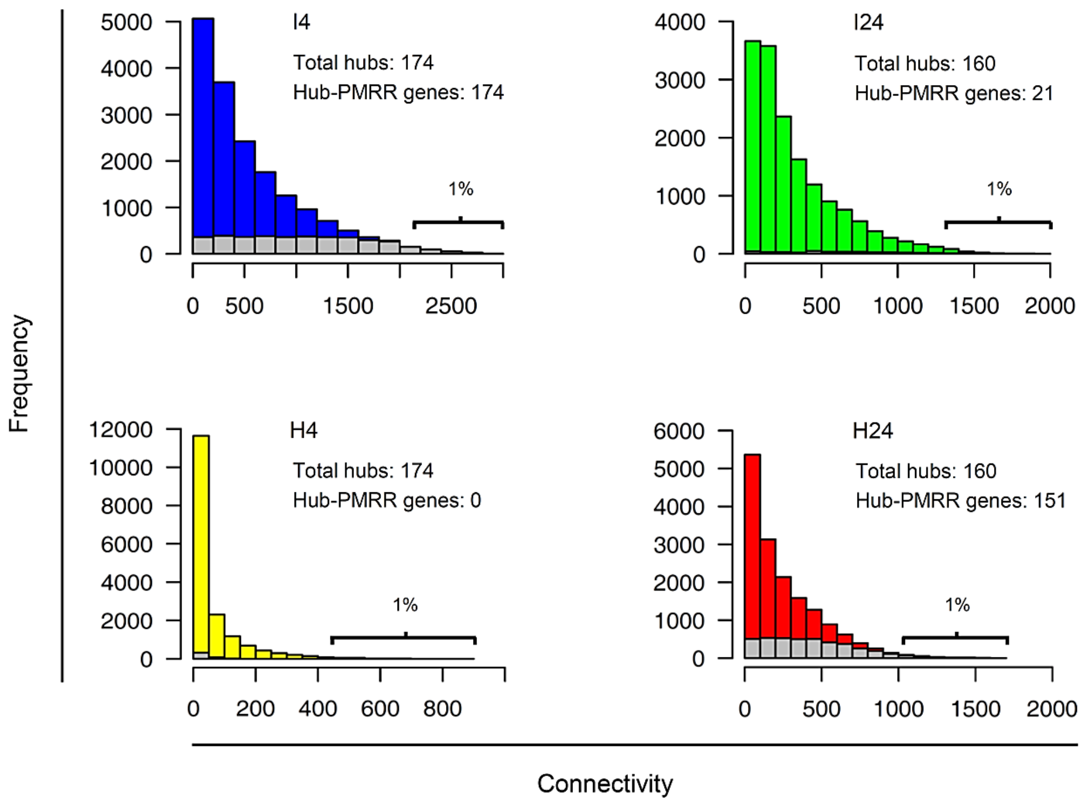

I4
Total hubs: 174
Hub-PMRR genes: 174
1%
5000
4000
3000
2000
1000
0
0
500
1500
2500
I24
Total hubs: 160
Hub-PMRR genes: 21
1%
4000
3000
2000
1000
0
0
500
1000
1500
2000
Frequency
H4
Total hubs: 174
Hub-PMRR genes: 0
1%
12000
10000
8000
6000
4000
2000
0
0
200
400
600
800
H24
Total hubs: 160
Hub-PMRR genes: 151
1%
6000
5000
4000
3000
2000
1000
0
0
500
1000
1500
2000
Connectivity

Figure S3

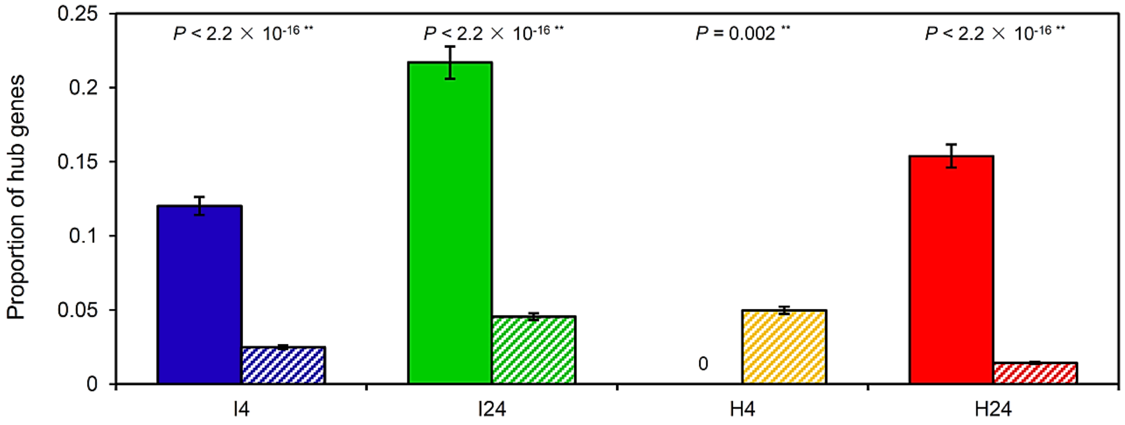
0.25
0.2
0.15
0.1
0.05
0
Proportion of hub genes
P < 2.2 × 10-16 **
P < 2.2 × 10-16 **
P = 0.002 **
P < 2.2 × 10-16 **
0
I4
I24
H4
H24

Figure S4

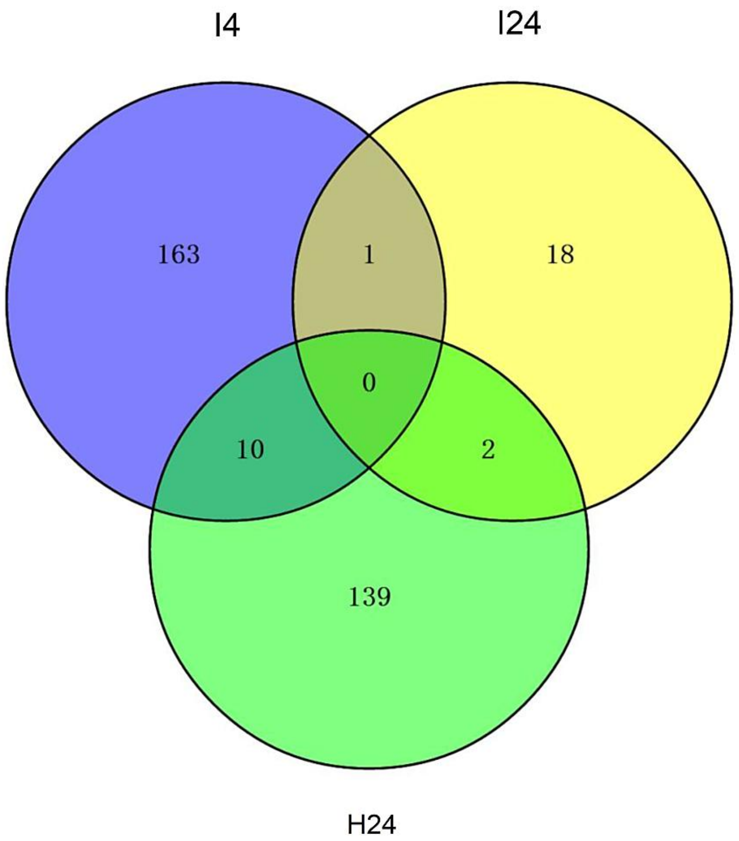

Figure S5

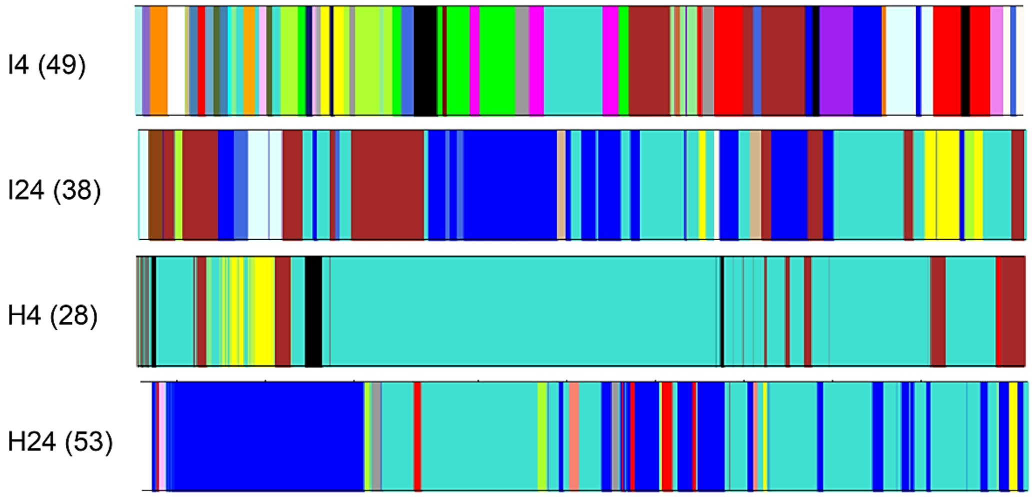

Figure S6

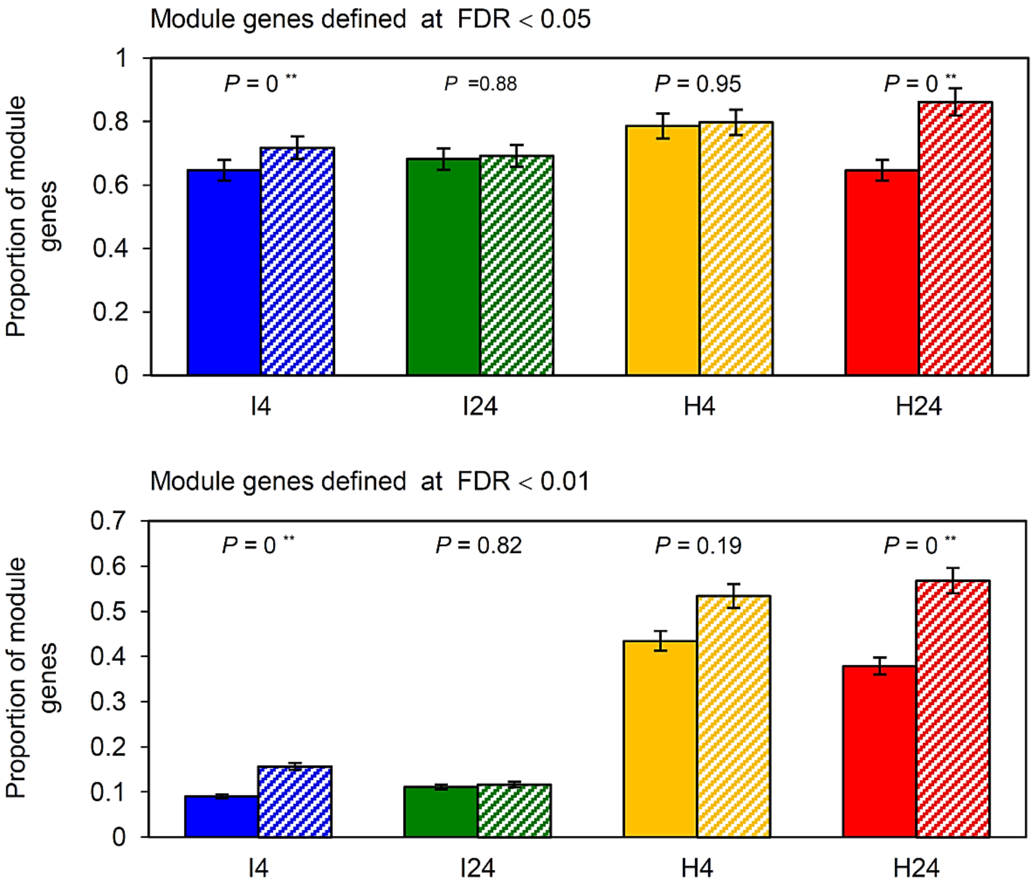
Module genes defined at FDR < 0.05
Proportion of module genes
P = 0 **
P =0.88
P = 0.95
P = 0 **
1
0.8
0.6
0.4
0.2
0
I4
I24
H4
H24
Module genes defined at FDR < 0.01
Proportion of module genes
P = 0 **
P = 0.82
P = 0.19
P = 0 **
0.7
0.6
0.5
0.4
0.3
0.2
0.1
0
I4
I24
H4
H24

Figure S7

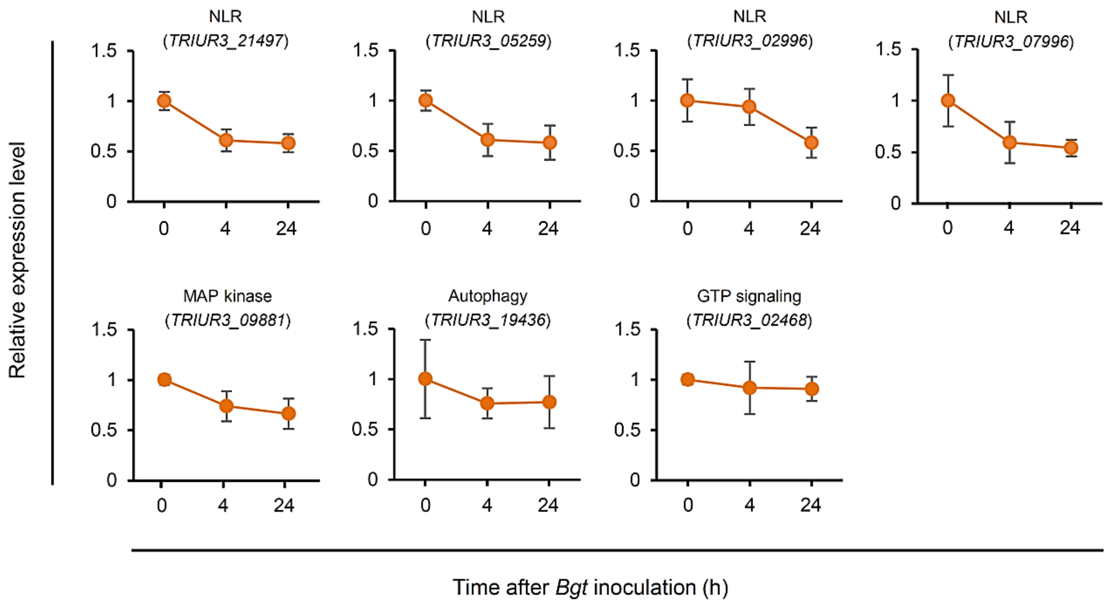

NLR
(TRIUR3_21497)
NLR
(TRIUR3_05259)
NLR
(TRIUR3_02996)
NLR
(TRIUR3_07996)
MAP kinase
(TRIUR3_09881)
Autophagy
(TRIUR3_19436)
GTP signaling
(TRIUR3_02468)
Relative expression level
1.5
1
0.5
0
0
4
24
Time after Bgt inoculation (h)

Figure S8

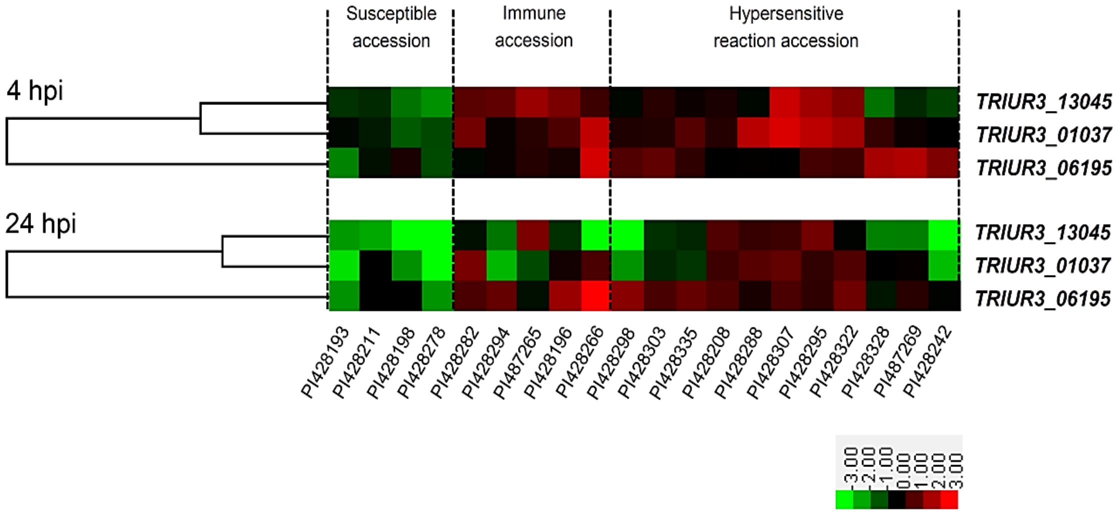
Susceptible accession
Immune accession
Hypersensitive reaction accession
4 hpi
24 hpi
TRIUR3_13045
TRIUR3_01037
TRIUR3_06195
TRIUR3_13045
TRIUR3_01037
TRIUR3_06195
PI428193
PI428211
PI428198
PI428278
PI428282
PI428294
PI487265
PI428196
PI428266
PI428298
PI428303
PI428335
PI428208
PI428288
PI428307
PI428295
PI428322
PI428328
PI487269
PI428242
-3.00
-2.00
-1.00
0.00
1.00
2.00
3.00

Figure S9

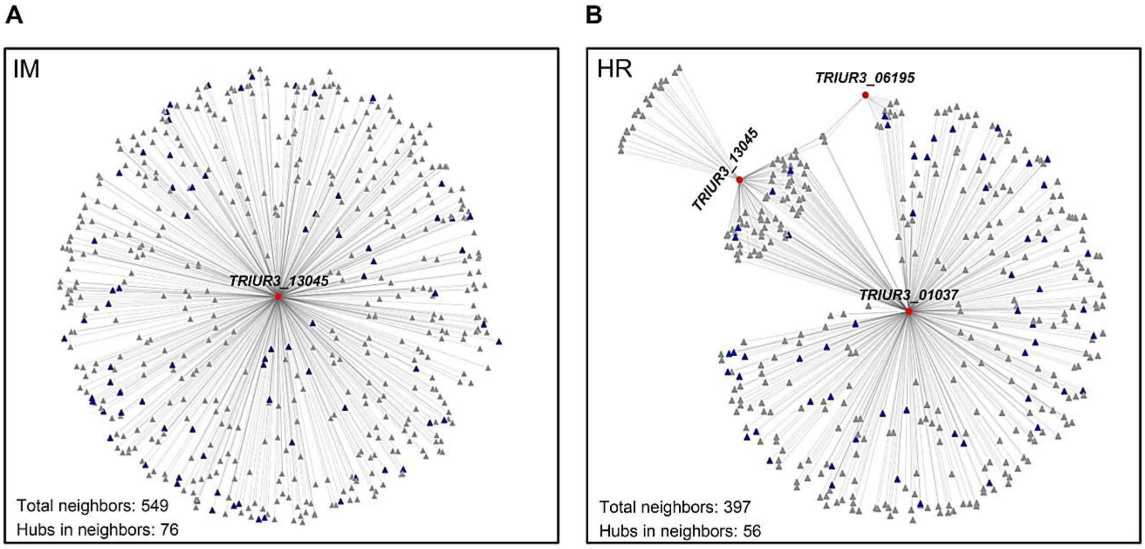
A
IM
TRIUR3_13045
Total neighbors: 549
Hubs in neighbors: 76
B
HR
TRIUR3_06195
TRIUR3_13045
TRIUR3_01037
Total neighbors: 397
Hubs in neighbors: 56
